# Automatic identification and characteristics analysis of crack tips in rocks with prefabricated defects based on deep learning methods

**DOI:** 10.1371/journal.pone.0327906

**Published:** 2025-07-15

**Authors:** Mingtao Gao, Minhui Li, Lu Chen, Zihao Guo, Chengyang Guo, Liping Li, Changsen Bu

**Affiliations:** 1 School of Emergency Technology and Management, North China Institute of Science & Technology, Langfang, China; 2 Key Laboratory of Mine Filling Safety Mining National Mine Safety Administration, North China Institute of Science & Technology, Langfang, China; 3 College of Energy and Mining Engineering, Shandong University of Science and Technology, Qingdao, China; 4 School of Qilu Transportation, Shandong University, Jinan, China; Shenyang Jianzhu University, CHINA

## Abstract

In complex geological environments, the morphology, orientation and distribution characteristics of cracks in the rock directly affect the stability assessment for rock masses and engineering safety decisions. However, the traditional manual interpretation method is inefficient and influenced by subjective factors, which makes it tough to fulfill the requirements for high-precision and automated detection. Especially in the rock specimen analysis of prefabricated multi-angle cracks, image quality and algorithm adaptability have emerged as the critical bottlenecks restricting the identification accuracy. For this reason, it is pressingly essential to realize high-precision and automatic identification in the crack tip of the rock. Firstly, in this study, SCB semi-circular disk specimens are exposed to three-point bending loading, which is sandstone with prefabricated cracks at 0°, 15°, 30°, 45° and 60°. The microsecond-level expansion process of multi-directional cracks is monitored by utilizing an ultrafast camera in the rock specimens. Secondly, three equalization methods are applied to the collected crack images of the rock specimens, including HE, AHE, and CLAHE, to enhance the accuracy of identifying cracks in the rock specimens. And the preprocessed crack images of the rock specimens are compared, which reveals the CLAHE method possesses the optimum preprocessing effect. Based on this, pixel-level annotations are performed on the pretreated crack images, and a dataset is established about cracks in the rock specimen at five different angles. The Deeplabv3 network and the U-Net network are adopted to build cracks recognition models of the rock specimen to predict and identify the crack tips on the rock. The final results demonstrate that the recognition accuracy of the U-net model is able to reach up to 99.4%, the precision is capable of amount to 97.3%, and the recall rate can attain to 95.6%, in the cracks identification of the rock sample with various angles. The recognition accuracy, the precision, and the recall rate of the U-net model have increased by 0.5%, 2.3%, and 4.3% respectively compared with the Deeplabv3 model. The research results provide new ideas for the intelligent detection of cracks in the rock mass, which offer high-confidence data support for engineering decisions in complex geological environments.

## 1 Introduction

Collapses occur frequently in rock engineering such as tunnel driving and mine exploitation, which is mainly induced by the development and expansion of cracks on the rock [1–3]. Due to the rock is a heterogeneous material under natural geological conditions, its interior and exterior are regularly accompanied by a large number of pores and micro-fractures. Abundant pores and micro-fractures of the rock seriously is influenced by the strength of rock mass structure [4–8]. With the increase of external force on the rock, the cracks in the rock gradually joins, expands and eventually become macro fracture, causing instability fracture of the rock mass. Among them, the fracture of rock is affected by its internal original defects, mineral crystal particle distribution, cementation state, external stress state, and boundary conditions. These combined effects make it difficult to distinguish the direction and distribution of the cracks on the rock. However, the fracture direction of the rock is an important factor in analyzing the stress state of the rock, and static loads will guide the direction of crack propagation. It is a significant need to clarify the crack tip, track the development and expansion process of the rock to prevent and control geotechnical disasters.

With the development of image processing technology [9–12], it provides a new possibility for crack identification by capturing crack images and combining computer analysis. At present, the methods for identifying cracks are mainly divided into two categories in combination with the computer technology. One is to extract and identify cracks through image processing technology [13–15]. For instance, Xu et al. [16] evaluated the confidence of candidate points through the eigenvalues of the Hessian matrix, symmetry, and gray values, proposed a crack detection algorithm for rocks based on confidence score. Jiang et al. [17] constructed a threshold adaptive segmentation algorithm by combining the Canny arithmetic and the OTSU method, achieving precise identification of striped cracks and irregular cracks. Su et al. [18] proposed a method grounded on the fractal theory for detecting surface cracks in concrete and masonry building structures, which is effectively reduce the influence of non-characteristic information on the crack identification results. Liang et al. [19] proposed a method dependent on digital image processing (DIP), which is enhanced the feasibility of measuring the crack evolution of asphalt mixtures. The other crack identification method is to automatically identify the crack images through the application of deep learning network models [20–24]. For example, Tang et al. [25] proposed a novel visual crack width measurement is proposed based on backbone double-scale features, which improves the automatic detection of cracks. Hu et al. [26] achieved high-precision 3D crack detection of structures through the fusion of high-precision LiDAR and cameras by extracting high-precision three-dimensional crack features. Meng et al. [27] suggested a pavement crack detection method founded on the You Only Look Once version 8 (YOLO v8) model, which is increased the generalization performance of the model and the effectiveness of crack detection. To automatically assess rock mass quality based on borehole core, Liu et al. [28] adopted a deep learning model to identify and calculate the dip angle of core cracks. Yuan et al. [29] improved the deep convolutional neural network (DCNN), took DeepLabv3+ as the overall framework, which is boosted a superior accuracy in identifying cracks. Cui et al. [30] proposed a deep residual attention convolutional neural network (DRACNN) for semantic segmentation of concrete cracks, which improved the performance of concrete crack classification. The above literature demonstrate the feasibility and reliability of applying image processing technology to identify cracks. However, the traditional image processing technology possesses human interference factors to identify cracks, which leads to limitations in the extraction of crack features. When the crack images are disturbed by complex backgrounds, uneven lighting, noise and other factors, traditional processing methods fail to satisfy the accuracy requirements for crack identification and it is difficult to detect cracks in coal and rock masses. Furthermore, due to the time resolution of the equipment not matching the speed of rock fracture, the fracture process of rocks be captured in real time, which leads to problems in the collection of the dataset. Moreover, the model training needs to rely on a large amount of data to prevent overfitting. Therefore, there is an imperative need for technologies and methods to track the crack tips of rocks in real time.

Most of the above-mentioned studies have adopted deep learning methods to intelligently identify cracks in materials such as concrete. However, whether the crack tip and the propagation process of cracks can be effectively detected in the ultrafast fracture process of SCB rock samples with different prefabricated crack angles at the microsecond level remains to be further explored. In this study, a picosecond pulsed laser was utilized as an optical shutter, combined with an ultrafast camera to capture the fracture process of rock in real time, and the microsecond-scale crack images of rock were subjected to recognition training. To enhance the applicability and accuracy of identification, the crack images of rock were obtained at angles of 0°, 15°, 30°, 45°, and 60° for further processing, respectively. Firstly, three equalization methods were adopted to process and improve the image detail information of different crack of rock with different precast angles, which facilitated image segmentation accuracy. Secondly, the Deeplabv3 model and the U-net model were employed to pre-train the datasets for the different crack angles of rock, and the weights were saved after pre-training. Then, the prediction results of the two models were evaluated respectively, the results indicated that the U-net model was more accurate in predicting the images of the crack tip. Ultimately, a prediction model was established, which was able to effectively identify the development and expansion in the cracks of rock at the microsecond level, based on the crack images of rock specimens with different prefabricated angles. The prediction model provides a new reference for the effective assessment and timely prevention of geological disasters.

## 2 Image preprocessing of rock sample fissure

The original images are collected by the Phantom VEO1010L ultrafast camera, which show the development and expansion of cracks in different prefabricated cracked rock specimens during the dynamic loading process. Due to the crack images of the rock specimen are affected by the environmental factors of the shooting, random noise is caused under long exposure. The noise blurs the edges of cracks in the image, which affects the recognition accuracy. To further improve the image quality, and extract the crack features of different prefabricated rock specimen images, it is imperative to perform filtering on the image to reduce the interference of noise on the feature information in the image.

### 2.1 Image Acquisition

**Fig 1:**
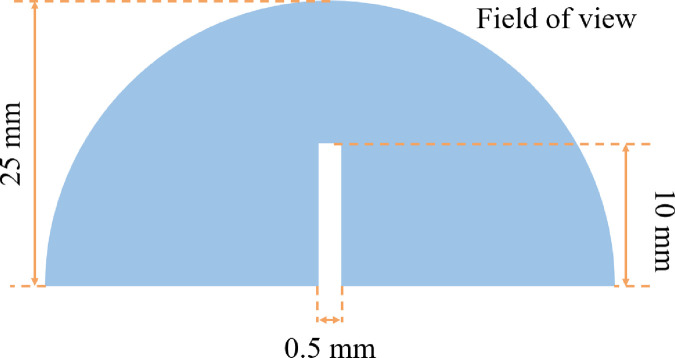
Rock specimen.

The experimental specimens: Standard semi – circular SCB rock specimens are adopted with a diameter of 50 mm. To more effectively observe the crack initiation and fracture propagation process in loaded rocks, pre-existing cracks of different angles were prepared at the midpoint of the rock samples, each namely 0°, 15°, 30°, 45° and 60°, with a width of 0.5 mm and a length of 10 mm. The rock specimen is shown in Fig 1.

**Fig 2:**
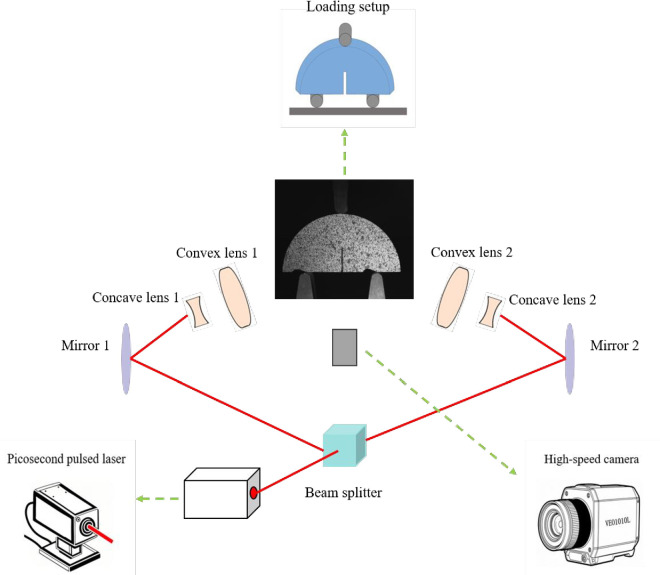
Experimental system setup.

Experimental setup: The experimental system consists of a picosecond pulsed laser source Sagittar-SLR, an E45.504 loading device manufactured by MTS, a spectrometer, a set of mirrors, a concave lens, a convex lens and a high-speed camera, as shown in Fig 2. The pulsed laser source is successively directed onto the rock specimen through a spectrometer, a reflector, a concave lens and a convex lens. To capture the entire fracture process with ultrafast temporal resolution, the frame rate of the high-speed camera is set to 100,000 frames per second. The wavelength of the picosecond laser is 532 nanometers, the repetition rate is 100 kilohertz, and the full width at half maximum (FWHM) of the laser is 15 picoseconds.

Experimental procedure: The functions of each component in the experimental system during operation are described as follows:

(1) The picosecond pulsed laser source generates the laser pulses used for irradiation.
(2) The beam splitter separates the laser into two beams of equal intensity.
(3) Mirrors 1 and 2 are employed to direct the laser beam.
(4) The plano-concave lens serves to expand the laser beam.
(5) Finally, the plano-convex lens collimates the expanded laser beam into parallel light.
(6) The loading device applies force to induce specimen failure.
(7) The high-speed camera captures the fracture process of the rock specimen, providing a dataset for crack tip modeling.

Laboratory Safety Regulations:

(1) Pay attention to the laser power. A higher pulse laser power can easily cause injuries to personnel. It is strictly forbidden for personnel to walk around at will and look directly at the emitted laser.
(2) During the experiment, phenomena such as debris ejection may occur during the rock fracture process. Experimenters should keep a certain distance from the experimental device.

Notes: The laser is triggered only once within a single exposure interval of the high-speed camera, and the specimen remains in darkness for the remainder of the time. Since the exposure time of the system is determined solely by the full width at half maximum (FWHM) of the pulsed laser, the time resolution can reach up to 45 picoseconds. In practice, the half-width at half-maximum (HWHM) of the pulsed laser serves as an optical shutter. The frame rate of the high-speed camera determines how many images can be captured during the fracture process.

### 2.2 Image equalization processing

The gray values of the cracks and edges are similar in the original image for the rock specimen, which makes distinguishing the boundaries of the cracks more complex. To obtain more detailed information of the images and enhance the contrast of the crack edges, it is indispensable to perform equalization processing on the filtered images. By redistributing the pixel values of the image through equalization, which is conducive to improving the ability of the model to extract crack features in the rock specimen. Three methods are selected to execute equalization processing on the images. Firstly, the Histogram Equalization (HE) method [31–33] is attempted to handle the crack images of the rock specimens. The HE is the most extensively used method in image enhancement applications. The HE enhances the overall clarity of the image by re-statistically distributing and allocating the gray-scale values of the original image through the cumulative distribution function. However, when the uniform background area is large, the HE method causes the local details of the image to be blurred. Therefore, the Adaptive Histogram Equalization (AHE) [34–36] method is adopted to conduct histogram equalization on each fixed-size local region in the crack images of the original rock specimens. The images with locally enhanced regions are stitched together, a smoothing method is applied to reduce the edge effect. However, the AHE method tends to overly amplify image noise. On this basis, the Contrast Limited Adaptive Histogram Equalization (CLAHE) [37–38] method is utilized to limit the frequency for gray-level pixel occurrence in the histogram of each local region. When the number of pixels in a certain area exceeds the set threshold, the excess part is evenly distributed to other gray levels, which avoids the distortion of the crack image in the original rock specimen caused by excessively high pixel contrast in some regions.

**Fig 3:**
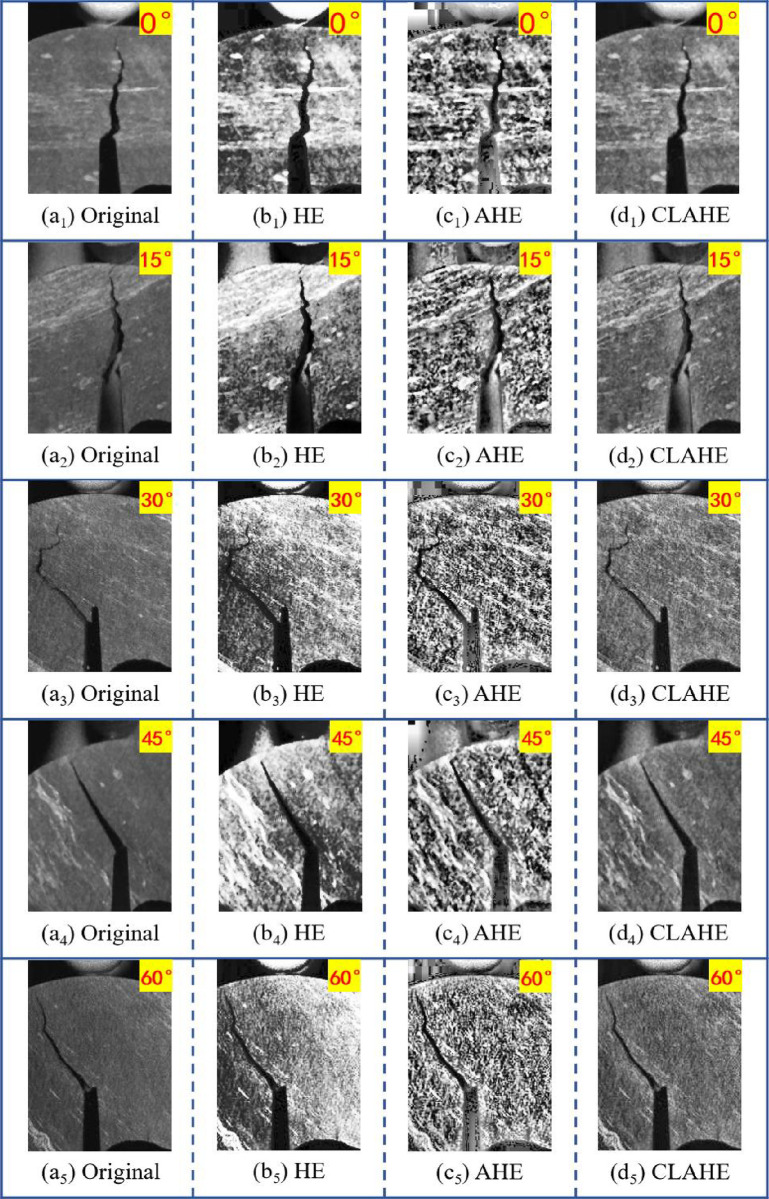
Results of different equalization methods.

In order to improve the quality for the crack images of the rock and facilitate the analysis of image segmentation tasks, three methods are applied to equalize the crack images of rock specimens at different angles such as 0°, 15°, 30°, 45° and 60°, including HE, AHE and CLAHE. The parameters of CLAHE are presented in Table 1, including an image size of 512 × 512 pixels, a tile grid size of 8 × 8, a total number of blocks equal to 64, and a clip limit set to 2.0. The results of the three equalization methods are shown in Fig 3.

**Table 1:** The CLAHE parameter settings.

Parameters	Numerical value
Image size	512 × 512
Tile grid size	(8 × 8)
Number of blocks	64
Clip limit	2.0

The HE is a global equalization method, which increases contrast by performing a global histogram mean operation on the crack images. However, as the rock contains other mineral components, such as organic matter and low-density minerals. By comparing the enhanced images of different crack angles in Fig 3, the global equalization of the HE method is able to cause misjudgment of other mineral components. The impurities around the crack are automatically equalized through the HE method, and the pixel value for the impurity becomes similar to the pixel value of the crack, which makes it difficult to accurately segment the crack tip in the rock. Meanwhile, the HE method fails to perform single equalization on local regions, the crack image exhibits an unsatisfactory processing effect on local details, which results in artifacts in the outcome. To address the aforementioned issues, the AHE method is employed to segment the entire image into regions, and execute individual equalization on each region of the different crack images in the rock specimens. The results in Fig 3 indicate that the problem of uncontrollable global equalization existing in HE is improved. However, AHE is a high sensitivity to random noise. In crack images of the rock under low light conditions, which may misjudges the trace noise in the low-contrast areas as the feature detail information of the image for enhancement, thereby introducing a large amount of noise and causing distortion in the balanced crack images. In addition, since AHE processes images in regions, improper setting of the region size is able to result in inconsistent boundaries when regions are reassembled at the end. And the transition in the detailed areas of the image is unsmooth, which causes interference to feature extraction in the task of crack image segmentation.

Owing to the drawbacks in the above two methods, this paper adopts the third equalization method for the crack images of the rock specimens, which is CLAHE. The CLAHE method introduces a contrast limit mechanism and interpolation technique on the basis of the AHE method. On the one hand, the CLAHE performs regional equalization on the crack images and limits the number of pixel values for each gray level within the region. According to the requirements for extracting rock specimen features, the CLAHE automatically adjusts pixel values and limit thresholds for cracks in the image locally. Concurrently, the CLAHE can effectively suppress the excessive amplification of noise and optimize the detail information in the crack region of interest within the image. On the other hand, interpolation techniques achieve a natural transition at region stitching boundaries through bilinear interpolation, which preserves the original image details. And bilinear interpolation has higher computational performance. Under the premise of ensuring the accuracy of the equalization processing, it avoids the appearance of artifacts in the equalization results and ensures no information is lost in the image during the equalization operation. By comparing three equalization methods, the CLAHE method possesses the superior effect on the processing of the crack images in the rock specimen. The CLAHE method is applied to enhance the rock specimens with different prefabricated crack angles in Fig 3. The results show that CLAHE is able to efficiently address the issue of noise amplification caused by image equalization, which adapts to brightness differences across different image regions. The CLAHE achieves contrast enhancement in the regions of interest and further refines details in the target boundary areas.

### 2.3 Preprocessing evaluation indicators

The peak signal-to-noise ratio (PSNR) and structural similarity (SSIM) are adopted to compare the advantages and disadvantages of the image processing effects for the three equalization methods. Among them, PSNR is a ratio between the maximum possible power of the signal and the power of the noise. Through comparing the pixel values of the image before and after preprocessing, the differences in pixel values of the image earlier and later are determined. The larger the PSNR value, the smaller the difference between the two images, and the better the image treatment effect.

The calculation process of PSNR is defined by:


$$PSNR=10\cdot \log_{10}\left(\frac{MAX_{I}^{2}}{MSE}\right)=20\cdot \log_{10}\left(\frac{MAX_{I}}{\sqrt{MSE}}\right)$$ (1)

where *PSNR* represents the peak signal-to-noise ratio, *MSE* denotes the mean square error, *MAXI* shows the maximum value of the image pixels, and *I* is the original image.

The SSIM is an index for measuring image similarity. SSIM compares preprocessed and unprocessed crack images from the perspectives of luminance, contrast, and structure. The larger the SSIM value, the greater the image similarity. The calculation formula of SSIM is expressed as:


$$SSIM=\frac{\left(2\mu_{x}\mu_{y}+C_{1}\right)\left(2\sigma_{xy}+C_{2}\right)}{\left(\mu_{x}^{2}+\mu_{y}^{2}+C_{1}\right)\left(\sigma_{x}^{2}+\sigma_{y}^{2}+C_{2}\right)}$$ (2)

where SSIM represents structural similarity, x and y are the crack images before and after equalization. $\mu_{x}$ indicates the average value of the crack image before equalization, $\mu_{y}$ shows the average value for the crack image after equalization, $\sigma_{x}$denotes the standard deviation of the fracture image before equalization, $\sigma_{y}$is the standard deviation for the crack image after equalization, and $\sigma_{xy}$ represents the covariance between the crack images before and after equalization. *C*_*1*_, *C*_*2*_, and *C*_*3*_ are constants.

**Fig 4:**
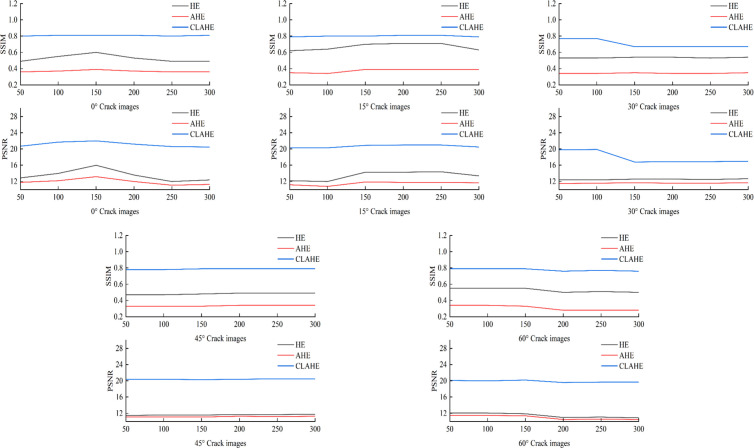
Comparison of Indicators for Different Equalization Methods.

The images of cracks from different angles are respectively processed by three equalization methods, which are HE, AHE, and CLAHE. And the PSNR values and SSIM values of the three methods are calculated to compare the advantages and disadvantages of the three equalization methods. The best method is selected for image processing of the crack images, and the results are shown in Fig 4.

By comparing the index results of the three methods in Fig 4, the PSNR and SSIM values corresponding to the CLAHE method are larger than those of the other two equalization methods. The PSNR value of CLAHE reaches about 20. The results show that after the CLAHE method is applied to the crack images, the difference is smaller between the original crack images and the processed images. Moreover, the images after equalization are less prone to distortion, which can better improve the accuracy of the model in crack recognition. The SSIM value of CLAHE is closer to 1, which indicates the original image is more similar to the crack image after CLAHE equalization. And the quality of the preprocessed images is improved, which facilitates the recognition of the vital features in the images by the model. The CLAHE is competent to enhance the local contrast of images, and more completely preserve the details and structural information in the images. The CLAHE makes the processed images perform better in both visual effect and quality assessment indicators, thereby improves the robustness of the subsequent crack tip identification model.

## 3. Establishment of rock specimen crack data set

Public datasets are unavailable for prefabricated crack images at different angles, and it is imperative to manually collect and establish the appropriate dataset. In this study, the dataset samples are established based on the pictures captured by high-speed cameras, which includes the crack propagation images of rock specimens with different prefabricated cracks. The angles of the prefabricated cracks are respectively set at five types: 0°, 15°, 30°, 45° and 60°. According to multiple angle types, 300 images are selected about cracks of the rock specimens for each angle, which ensures the crack edges are clear and the angles distinct in each image. To establish a high-accuracy crack identification model of the rock specimen, high-quality crack images are manually selected for preprocessing. The final determined dataset contains a total of 1,500 crack images in the rock specimen from five different angles, and the dataset is randomly divided into a training set and a test set in a ratio of 7:3. The dataset consists of 1050 images for the training set and 450 images for the test set. The specific division of the dataset samples is shown in Table 2.

**Table 2:** Data sets of rock specimens with different prefabricated crack angles.

Cracks from different angles	Training set sample number	Test set sample number
0°	210	90
15°	210	90
30°	210	90
45°	210	90
60°	210	90

**Fig 5:**
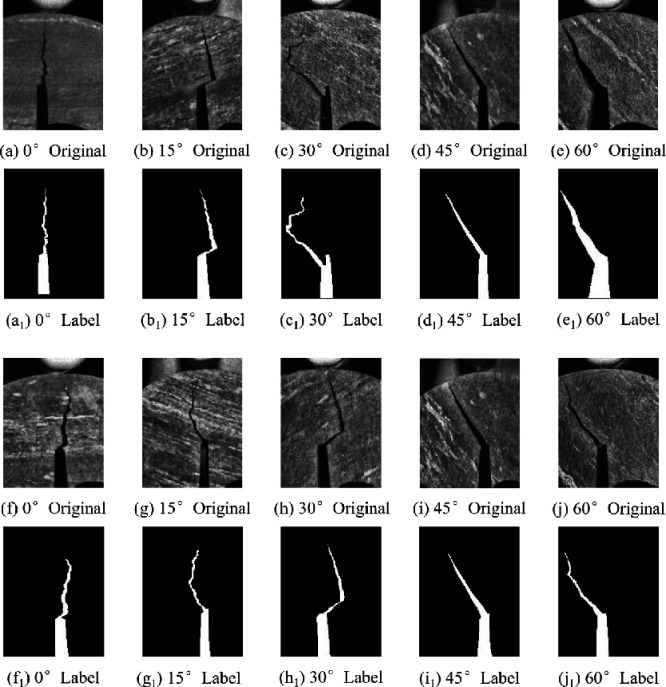
Partially annotated images.

The image processing software Labelme is utilized to manually annotate the pixels in the crack images from different angles and generate the relevant label images. The labeled images provide data for subsequent model training to accurately identify the morphology, length, width and location of cracks in the rock samples. A portion of the labeled images are shown in Fig 5. The label values are divided into two categories: cracks and background. The crack areas are marked in white, and the non-crack areas are marked in black.

## 4. Establishment of rock specimen crack identification model

### 4.1 DeepLabv3 network model

**Fig 6:**
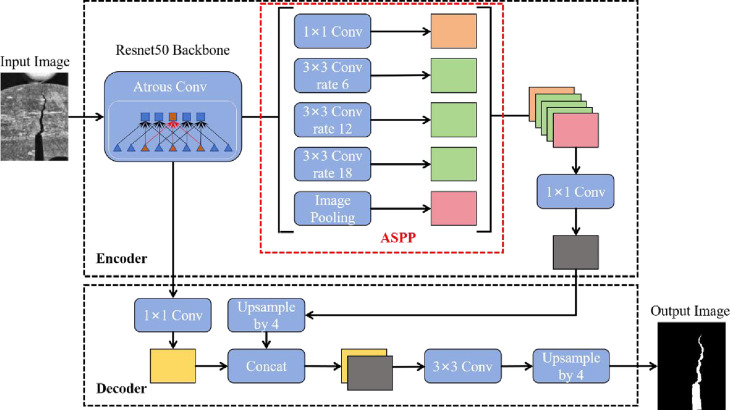
Deeplabv3 network model structure.

DeepLabv3 [39–41] is a deep learning model for semantic segmentation proposed by Google. The structure of the DeepLabv3 model is shown in Fig 6. The DeepLabv3 network model is structured as an encoder and a decoder. The encoder part includes backbone network feature extraction and the Atrous Spatial Pyramid Pooling (ASPP) module to achieve multi-scale feature extraction of the image. The decoder concatenates the feature maps, which are extracted by the backbone network and processed by the Atrous Spatial Pyramid Pooling module. The decoder fuses multiple sets of features at different scales to enhance the accuracy of the model in recognizing the target image.

Resnet50 is used as the backbone network for the feature extraction part of the backbone network. Resnet50 has a relatively deep network structure, which reduces the problem in gradient vanishing during the training for crack images through the connection of residual blocks. Resnet50 is able to mine the feature information of crack images from shallow to deep. The low-level convolutional layers can extract features like texture and edge of rock specimens, the high-level convolutional layers can extract features like the length and shape of the crack. The Atrous Convolution (Atrous Conv) is introduced into the encoder of the Deeplabv3 model. On the one hand, it is unable to alter the resolution of the original image and refine the detailed information. On the other hand, the Atrous convolutions with different dilation rates are capable of extracting crack features at different levels. The ASPP module is included in the decoder section, which consists of four parallel Atrous convolutions and one pooling layer. The ASPP model utilizes the Atrous convolutions with different dilation rates to extract multi-level features from the image, and then employs a global pooling layer to obtain the information of the entire crack image. The decoder concatenates the crack feature images of different dimensions and different levels under the convolution operations with different dilation rates and the global pooling operation. Combining micro and macro features, diverse crack characteristics are provided for subsequent model training input, which boosts model performance in image segmentation and crack tip recognition.

The decoder part first applies a 1 × 1 Conv operation to the image after the Atrous convolution in the feature extraction of the backbone network. The purpose of the convolution operation is to perform dimensionality reduction on the feature map of the output crack, which ensures better feature blending. Secondly, the feature map output by the ASPP module is up-sampled to scale the image size back to the original input size, preserving the original information. Finally, the decoder achieves precise identification of the images on the crack tips by fusing the crack features extracted from the backbone network and the ASPP module.

### 4.2 U-net rock sample fracture recognition model

**Fig 7:**
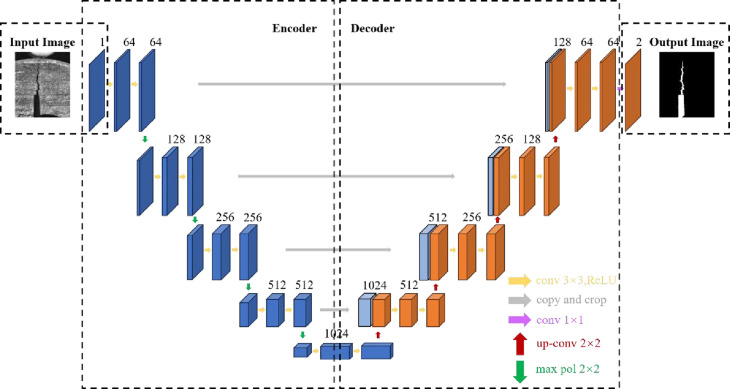
U-net network model structure.

The construction of the U-net network model is similar to a U shape [42–45]. The U-Net network model consists of two parts, which are the encoder on the left and the decoder on the right. The encoder extracts feature through convolutional layers and then executes sparse processing on the feature maps by down-sampling to reduce the amount of data operations. The decoder restores the spatial structure information of the original image through transposed convolution, and up-sampling is employed to increase the size of the feature map to a higher level, which adds more detailed information to the feature map. The same-level layers are concatenated through skip connections, connecting the corresponding feature maps in the encoder and decoder. Concatenating feature maps enables the decoder to better utilize the feature information at different levels, which improves the accuracy and detail retention ability of image segmentation. The structure of the U-net network model is shown in Fig 7.

The left part in Fig 7 is the encoder, the backbone feature extraction network, which includes convolution operations and pooling operations. The encoder sets the convolution kernel size based on the crack image characteristics and extracts features by sliding the convolution kernel on the original crack image. Two consecutive convolution operations are carried out to enhance the extraction ability of crack features. After each convolution operation, the ReLU activation function [46] is employed to integrate nonlinear problems and improve the learning ability of the model for complex nonlinear problems. The pooling operation in the encoder adopts a max pooling layer. The pooling operation is capable of highlighting the most significant features in an image by selecting the maximum value within a local region as the output. In the crack images, the significant features requiring identification, such as the edges, textures and contours of the cracks, possess relatively large pixel values. The max pooling operation can retain features with larger values while ignoring other relatively minor information, reducing data redundancy and precisely capturing the crack features in the image, which effectively enhances the ability of the model to identify cracks. The backbone feature extraction network part conducts feature extraction from shallow to deep on the original crack image through the above-mentioned convolution operations, activation functions, pooling operations, etc. The backbone feature extraction includes features such as the texture, crack edges, crack angles, and sizes of the crack image, which provides a foundation for the U-net model to accurately predict crack tips in the end.

The ReLU activation function formula is expressed as:


$$y=\max(0,x)=\left\{ \;\;\;\begin{array}{c} x(x>0)\\ 0(x\leq 0) \end{array}\right.$$ (3)

where x represents the input value and y is the output value.

To clearly identify the edge of the crack, further operations are performed on Eq. (1), resulting in Eq. (2).


$$\frac{\partial y}{\partial x}=\left\{ \;\;\;\;\begin{array}{c} 1(x>0)\\ 0(x\leq 0) \end{array}\right.$$ (4)

The right half of Fig 7 is the decoder, the enhanced feature extraction network, which includes up-sampling, skip connections, convolutional blocks and the output layer. The up-sampling part employs bilinear interpolation to restore the feature map extracted by the backbone to the original size of the crack image, which prevents the loss of detailed information in the original crack image and avoids overfitting issues in the subsequent training process. After the up-sampling operation, a set of deep-level crack feature maps is obtained. The deep-level crack feature maps are concatenated with the corresponding shallow-level crack feature maps in the encoder through skip connections to achieve crack image feature fusion. Ultimately, convolution operations are continuously applied to the concatenated feature maps to combine the local crack feature information into higher-level features. The image segmentation categories in this article include cracks in the rock specimens and background. The output layer employs a 1 × 1 convolution operation to adjust the feature map channels. Without altering the crack feature image, the convolution operation converts the prediction image channels to the corresponding number of categories. The Softmax function [47] is utilized to convert the output layer values into probabilities corresponding to the two categories of the cracks in the rock specimen and background, enhancing model classification accuracy. The enhanced feature network extraction part is applied to fuse multi-scale features, which makes the feature representation incorporate both large-scale abstract features and small-scale specific features. Feature fusion enables the U-net model to accurately identify and segment the crack tip of the rock specimen targets.

The Softmax function formula is defined as:


$$S\left(x_{i}\right)=\frac{e^{x_{i}}}{\sum {\mathrm{\backslash nolimits}}_{i=1}^{n}e^{x_{i}}}$$ (5)

Where $S\left(x_{i}\right)$ indicates the i-th element of the output of the Softmax function, *i* represents the i-th element in the input vector, *n* signifies the number of categories, and *e* is the natural constant.

The dataset is utilized as the input data for the U-net network model, which includes images of cracks from different angles. Firstly, the dataset is normalized to facilitate the improvement of the model training effect. Secondly, the normalized data is input into the encoder part of the U-net model. As the convolution and pooling operations increase continuously, features of the crack images at different scales are extracted, such as the shape, texture, and position of the cracks. Then, through the up-sampling operation in the decoder of the model, the feature image of the crack is restored to the original image size. After each up-sampling operation, the corresponding feature map of the encoder demands to be spliced. The feature information at different levels is parsed by the decoder, and then the convolution operations are used to fuse the concatenated feature images to improve the accuracy of the U-net network model in identifying the images of crack tips. Finally, a 1 × 1 Conv operation is employed to change the number of channels in the predicted crack image to 2. The output predicted image includes the non-crack area and the crack area, which corresponds to the two channels of the output image. By comparing the input original crack image with the output recognition prediction image, a reference is provided for evaluating the recognition performance of the model.

### 4.3 Comparison of model training loss functions

In this investigation, the U-net network model and Deeplabv3 network model are adopted to achieve the identification of pre-existing crack tips at different angles in rocks. In image segmentation, the output is a binary classification task, including the crack area and the non-crack area. The loss function is mostly defined by the cross-entropy loss function suitable for binary classification tasks [48]. The true labels in image segmentation and the predicted image labels by the model are regarded as two probability distributions, which are the true probability and the predicted probability. and the predicted image labels by the model represent the predicted probability. The cross-entropy loss function assesses the accuracy for the predictions of the model by calculating the difference between the true probability and the predicted probability distribution. The smaller the value of the cross-entropy loss function, the more stable the model training effect and the more accurate the training results. The cross-entropy loss function is defined as follows:


$$L=-\frac{1}{n}\sum_{i=1}^{n}\left[x_{i}\log(a_{i})+(1-x_{i})\log(1-a_{i})\right]$$ (6)

Where *L* shows the cross-entropy loss function, *n* represents the number pixels in the image, *x*_*i*_ is the true label of the i-th pixel, the non-crack region is 0, the crack region is 1, and *a*_*i*_ indicates the probability value of the i-th pixel predicted as the target, in the interval [0,1].

**Fig 8:**
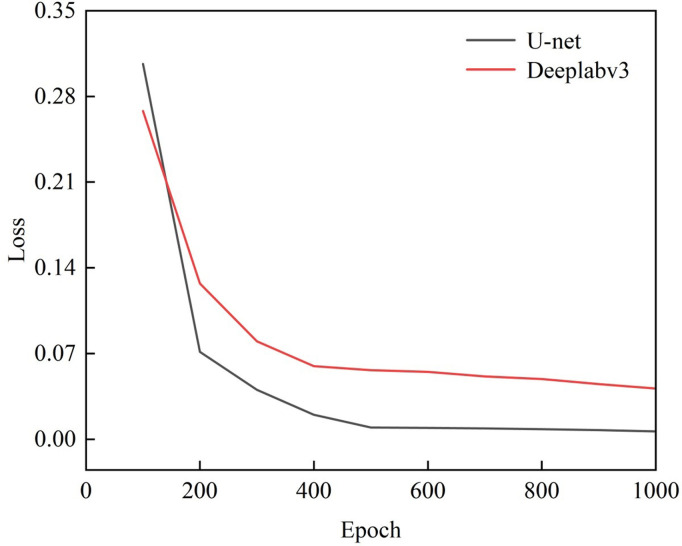
Training loss function curve.

The U-net model and Deeplabv3 model are used to pre-train the images of cracks at 0°, 15°, 30°, 45° and 60°. The model training environment and training parameter settings are shown in Table 3. The NVIDIA RTX 3070 GPU, Windows 10 system, PyTorch 1.10.2, CUDA 12.6, CUDNN 8.2.0, and Python 3.8.5 are selected as the model training environment. The training parameter settings include an optimizer as RMSprop, a weight-decay of 1e-8, a momentum is 0.9, and a shuffle seed number of 42. Concurrently, the training rounds are set to 1000 epochs, with a batch size of 2 and a learning rate of 0.0001. During the training process, the loss value is recorded every 100 epochs. The cross-entropy loss function curves are shown in Fig 8, which include the two models for predicting the crack images from different angles.

**Table 3:** Model training environment and training parameter setting table.

Parameters	Settings	Environment	Version
Weight‐decay	1e-8	GPU	NVIDIA RTX 3070
Momentum	0.9	CUDA/CUDNN	V12.6/V8.2.0
Shuffle seeds	42	Python	V3.8.5
Optimizer	RMSprop	Pytorch	V1.10.2

As shown in the above Fig 8, with the increase of the number of iterations, the values of the cross-entropy loss function are constantly decreasing during the training process of both the U-net model and the Deeplabv3 model. The overall trend shows a rapid decline at first, followed by a slow decline, and finally tends to be horizontal. In the cross-entropy loss function curves corresponding to the two models, it can be observed the cross-entropy loss function value of the U-net model decreases at a faster rate as the number of training iterations increases. When the number of training iterations reaches 500, the loss value has already dropped to 0.01, and the convergence process takes less time. In contrast, the loss function curve of the Deeplabv3 model decreases slowly during the training process and gradually converges only after 600 training iterations. Once the training times reach 1000, the loss function value of U-net model stabilizes at about 0.01, and the loss function value of the Deeplabv3 model stabilizes at about 0.05. The loss value of the U-net model is smaller than that of the Deeplabv3 model, which indicates that the predicted crack tip results of the U-net model are closer to the real crack labels. Therefore, the pre-training effect of the U-net network model is significantly superior to the Deeplabv3 model on the images of crack tips in the rock from different angles.

## 5 Analysis of rock sample fracture identification results

### 5.1 Analysis of model recognition results

**Fig 9:**
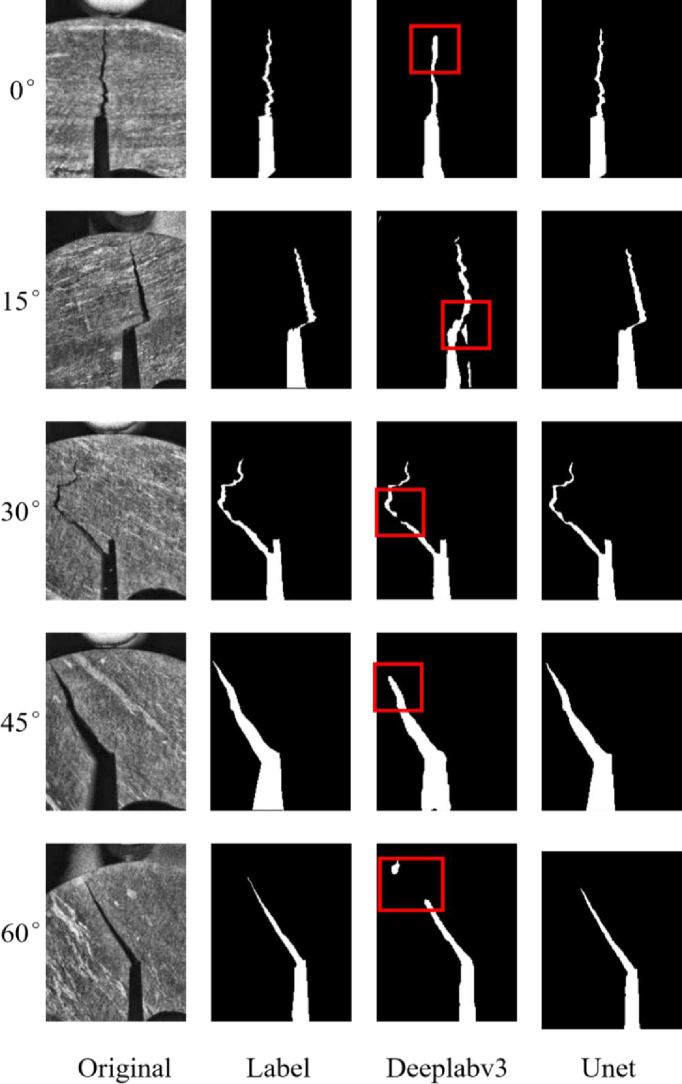
Visual comparison of fracture identification by different models.

To analyze the recognition effect of the U-net model on crack tips in the rock specimens at different angles, the Deeplabv3 model and the U-net model are respectively applied to test and verify the images in the test set, and the prediction results of the two models are saved. A set of corresponding images are randomly selected for comparison about the predicted results of the prefabricated crack tips in the rock from different angles, as shown in Fig 9. The figure includes original crack images from different angles, the corresponding real crack label images, the prediction result images of the Deeplabv3 model, and the prediction result images for the U-net model.

As depicted in Fig 9, which illustrates the comparison of crack tip identification results for rock specimens at five distinct angles, the crack tip identification results generated by the U-net model demonstrate a higher degree of similarity to the actual labeled crack images. First, survey the identification results of the crack tips by the Deeplabv3 model. As illustrated in the figure, for the crack image of the rock at 0°, the identified crack tips exhibit smooth boundaries, and the notched features in the original image remain unrecognized. Consequently, the Deeplabv3 model demonstrates limitations in accurately identifying the notched tips of 0° cracks in the rock. For the 15° crack image in the rock, two incorrect predictions are observed on the left side of the crack prediction result image, where non-crack regions are misclassified as crack regions. The demonstrates that the Deeplabv3 model exhibits prediction inaccuracies when identifying 15° cracks of the rock. For the crack image of the rock at 30°, it is clearly observable that a small portion of the crack region in the predicted image is not successfully identified, leading to incomplete crack delineation. The Deeplabv3 model possesses apparent defects in the prediction of 30° cracks in the rock. For the 45° crack images in the rock, the prediction outcomes exhibit similarities to the 0° crack images of the rock, and neither achieves precise identification of the crack tips. For the crack images in the rock at 60°, the prediction results display both incorrect pixel-level classifications and incomplete crack tips delineation. Overall, the Deeplabv3 model holds certain issues in predicting crack tips of the rock at different angles. Secondly, through analyzing the recognition outcomes of the U-net model for images at crack tip in the rock specimens at various angles, it is evident that the U-net model can precisely identify the tips of cracks. Moreover, there are no instances of incomplete crack recognition or misclassification of crack regions. Therefore, compared with the Deeplabv3 model, the U-net model reveals a higher accuracy in identifying crack tips of the rock from different angles. Additionally, it exhibits significant improvements in recognizing detailed features such as edges and corners within the image, which gets closer to the real crack image.

### 5.2 Model evaluation metrics

To more effectively evaluate the segmentation capability of the two models on images about crack tips at different angles, the article adopts accuracy, precision, recall, F1-score and MIoU as the indicators for model recognition assessment.

The confusion matrix is employed as a tool for assessing the performance of models in binary classification problems. In the confusion matrix, the segmentation effects of the two models on the target area tend to be visually contrasted in terms of the advantages and disadvantages. The confusion matrix is adopted to exhibit the correspondence between the true labels and the predicted labels, which objectively assesses the performance of the model. Subsequently, the confusion matrix is employed to define four indicators to render the effectiveness evaluation of the model more persuasive, such as accuracy, precision, recall, and F1 score. For binary classification problems, the confusion matrix is typically divided into true positives (TP), false positives (FP), true negatives (TN), and false negatives (FN).

Pixel Accuracy (PA), which represents the proportion of both positive and negative samples correctly predicted in the total number of samples. The calculation formula for PA is defined as:


$$PA=\frac{TP+TN}{TP+TN+FP+FN}\times 100\;\%$$ (7)

Where *PA* represents the accuracy rate, *TP* stands for the number of positive samples correctly predicted as the positive class by the model, *FP* indicates the number of negative samples incorrectly predicted by the model as the positive class, *TN* is the number of negative samples correctly predicted by the model as the negative class, and *FN* denotes the number of positive samples incorrectly predicted by the model as the negative class.

Precision (P) denotes the proportion of true positive samples among the predicted positive samples. The calculation formula for P is expressed as:


$$P=\frac{TP}{TP+FP}\times 100\;\%$$ (8)

Where *P* signifies the precision rate.

Recall (R) indicates the proportion of samples with true positive values predicted as positive samples. The calculation formula for R is as follows:


$$R=\frac{TP}{TP+FN}\times 100\;\%$$ (9)

Where *R* denotes the recall rate.

The F1 score (F1) is a comprehensive measure of recall and precision, which can more comprehensively reflect the crack identification effect of the model. The calculation formula for the F1 is defined as:


$$F1=2\times \frac{P\times R}{P+R}\times 100\;\%=\frac{2TP}{2TP+FP+FN}\times 100\;\%$$ (10)

Where *F1* is the F1 score.

The larger the values of the four evaluation indicators, accuracy, precision, recall, and F1 score, the more excellent the segmentation effect on the crack images and the nearer the predicted cracks in the rock specimens are to the actual cracks.

Intersection over Union (IoU) is an indicator employed in image segmentation tasks to measure the degree of overlap between the predicted results and the true results. The Mean Intersection over Union (MIoU) is the average of the Intersection over Union (IoU) for all categories. MIoU is applied to wholly evaluate the performance of a model in multi-class tasks. The MioU value ranges from 0 to 1. The closer the value is to 1, the closer the predicted crack results of the model are to the true crack labels, which indicates better model performance and more accurate identification of cracks at different angles in rock specimen images. Conversely, the closer the value is to 0, the poorer the prediction of the model effect on cracks in the rock specimen. The expressions of IoU and MioU are as follows:


$$IoU=\frac{\left|P⋂Q\right|}{\left|P⋂Q\right|}$$ (11)

$$MioU=\frac{1}{n+1}\sum_{n=0}^{n}\frac{\left|P_{n}⋂Q_{n}\right|}{\left|P_{n}⋃Q_{n}\right|}$$ (12)

Where *IoU* stands for Intersection over Union, *MioU* denotes Mean Intersection over Union, *n* represents the number of non-background classes, *P* is the label image, and *Q* is the predicted image.

**Fig 10:**
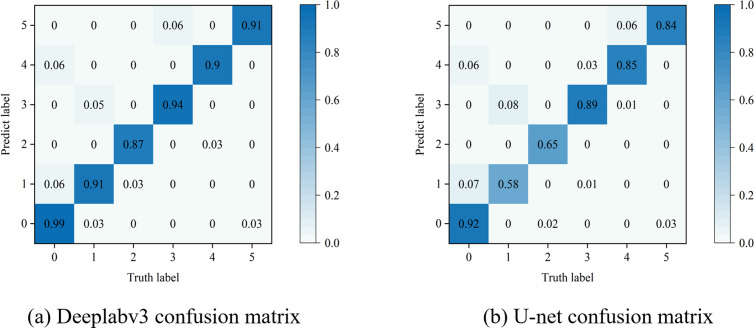
Confusion matrices of different models.

In this study, two models are adopted to identify crack tips in rock specimens from diverse angles. Essentially, it is a six-classification task of images. The non-crack area (background) is set as the first category, 0° cracks as the second category, 15° cracks as the third category, 30° cracks as the fourth category, 45° cracks as the fifth category, and 60° cracks as the sixth category. The rows of the confusion matrix represent the true categories, and the columns represent the predicted categories. The confusion matrices corresponding to the prediction probabilities of different categories by the U-net model and the Deeplabv3 model are shown in Fig 10.

Fig 10(a) shows the confusion matrix relevant to the Deeplabv3 model. The prediction accuracy of the Deeplabv3 model exists discrepancy for different categories varies. The Deeplabv3 model owns the lowest prediction accuracy for 0° crack tips in the rock, at only 58%, and the recognition accuracy of the background by the Deeplabv3 model is only 92%. Moreover, the Deeplabv3 model possesses more cases of misclassified samples. Fig 10(b) illustrates the confusion matrix corresponding to the U-net model. The accurate prediction probabilities of the U-net model are 99%, 91%, 87%, 94%, 90% and 91% respectively for the identification of the crack tips in the rock in the background, 0°, 15°, 30°, 45° and 60°. The prediction accuracy of the U-net model exceeds 87% for each category, and the prediction accuracy reaches 99% for non-cracked areas. The results show that for the prediction of non-cracked areas and crack tips in rock specimens at five different angles, the U-net model possesses a greater prediction accuracy probability than the Deeplabv3 model. The U-net model is more stable in identifying crack tips from various angles and boasts a superior accuracy rate in category recognition. The U-net model demonstrates superior applicability in the domain of identifying prefabricated crack tips within rock specimens across diverse angles.

To mitigate the potential bias introduced by the limited size of the dataset in model outcomes, the generalization ability of the U-net model and the Deeplabv3 model was verified by using the five-fold cross-validation method. Five-fold cross-validation was conducted for each angle of the crack. By analyzing the cross-verification results of the two models, it is evident that the U-net model demonstrates stable recognition accuracy of approximately 99% for crack tips at various angles. In contrast, the recognition accuracy of the Deeplabv3 model for crack tips at different angles varies between 86.11% and 99.13%. The results indicate that the U-net model has a excellent and stable prediction effect on the prefabricated crack tips at different angles.

To further verify the ability of the U-net model to identify crack tips at different angles, the Deeplabv3 model and the U-net model are compared. The values of the two models and their related various evaluation indicators are shown in Table 4.

**Table 4:** Comparison of evaluation index values of the two models.

Model	Angle	PA	P	R	F1	MioU
U-net model	0°	99.3%	96.1%	92.3%	94.1%	93.8%
	15°	99.3%	97.3%	95.6%	96.4%	96.7%
	30°	99.0%	91.4%	88.7%	90.0%	90.4%
	45°	99.4%	96.2%	93.0%	94.5%	94.5%
	60°	99.2%	96.6%	92.5%	94.5%	94.6%
Deeplabv3 model	0°	86.7%	80.6%	79.5%	80.0%	84.9%
	15°	94.7%	91.8%	90.1%	90.9%	77.5%
	30°	98.7%	90.9%	88.7%	89.7%	87.2%
	45°	98.2%	95.8%	90.9%	93.2%	86.5%
	60°	98.9%	95.0%	91.3%	93.1%	92.4%

As shown in Table 4, the U-net model achieves maximum values of 99.4%, 97.3%, 95.6%, 96.4%, and 96.7% for the PA, P, R, F1, and MioU metrics, respectively. In contrast, the Deeplabv3 model attains corresponding maximum values of 98.9%, 95.0%, 91.3%, 93.1%, and 92.4%. Compared with the Deeplabv3 model, the U-net model has increased by 0.5%, 2.3%, 4.3%, 3.3%, and 4.3% respectively. The more considerable the values for each evaluation metric, the superior the model recognition effect. Meanwhile, the U-net model outperformed the Deeplabv3 model in all evaluation metrics for crack recognition of the rock in the identification of crack tips at 0°, 15°, 30°, 45° and 60°. The numerical results show that the U-net model can maintain relatively stable crack identification performance in the identification of crack tips in rock specimens at different angles. When confronted with various complex image scenarios, such as changes in lighting conditions, shooting angles, and image resolutions, the U-net model achieves precise recognition and segmentation of the target.

From the model evaluation index results Table 4 presented in the manuscript, it can be observed that the U-net model achieves an average accuracy (PA) of 99%, while the Deeplabv3 model attains an average accuracy (PB) of 95%. First, the combined accuracy (P) of the two models is calculated, yielding P ≈ 0.9787. Subsequently, the standard error (SE) is computed using the combined accuracy (P), resulting in SE ≈ 0.0081. Next, the Z statistic is determined by incorporating PA, PB, and SE, leading to Z ≈ 4.81. Finally, the critical value Z_*α*/2_ corresponding to the 95% confidence level (*α* = 0.05) is compared with the calculated Z statistic. Given that the critical value Z_*α*/2_ = ±1.96 and the computed Z ≈ 4.81 significantly exceeds this threshold, it can be concluded that the difference in accuracy between the U-net model and the Deeplabv3 model is statistically significant.

## 6 Conclusions

To explicitly determine the distribution of cracks in the rock and the propagation directions under varying stress conditions, while addressing the limitations of traditional crack detection methods such as high subjectivity and insufficient contrast. Automated recognition is implemented, which captures the crack tip with high precision and multiple angles. The article analyzes the crack evolution images of SCB semi-circular disc sandstone specimens with prefabricated cracks at 0°, 15°, 30°, 45° and 60° during the three-point bending loading process. The main conclusions drawn are:

(1) Three equalization methods are adopted to preprocess the images of the crack evolution process for rock specimens with different prefabricated cracks, such as HE, AHE, CLAHE. The values of the equalization index parameters PSNR and SSIM are compared. The PSNR value of CLAHE reaches around 20, and the SSIM value is closer to 1. The results indicate that the equalization effect of CLAHE is the optimum.
(2) To establish an automatic rock crack identification model, the U-net model and Deeplabv3 model are respectively selected to analyze the crack tips of rock specimens with different prefabricated angles. The PA, P, R, F1, and MioU metrics of the U-net model can reach up to 99.4%, 97.3%, 95.6%, 96.4%, and 96.7% respectively, with all metric parameters being greater than the Deeplabv3 model. The results demonstrate the efficiency of the U-net model in the task for identifying crack tips from diverse angles.
(3) The recognition results of the U-net model and the Deeplabv3 model are compared for randomly selected images of cracks at different angles. The recognition images show that the U-net model outperforms the Deeplabv3 model in recognizing non-crack areas and the images of crack tip at 0°, 15°, 30°, 45° and 60°.

Natural rock masses typically exhibit a variable number of cracks in various sizes. The process of crack propagation within the rock masses demonstrates pronounced anisotropic characteristics. Under stress perturbation, the cracks of rock exhibit mutual interaction and potential coalescence, which may result in the overall instability of the rock mass and ultimately lead to geotechnical engineering failures. The intelligent identification technology for rock fractures is capable of rapidly and precisely determining the distribution of fractures, quantifying the scale, orientation and connectivity of cracks. Moreover, the intelligent identification of rock cracks can supply extensive high-precision datasets for rock mechanics research, thereby facilitating a more comprehensive understanding of fracture propagation and rock mass failure mechanisms. This study employs the U-net model to identify crack tip images during the ultrafast fracture process of rocks. While the recognition of tiny cracks remains a challenge. The next step involves incorporating an attention mechanism into the model to automatically extract the most salient features for crack identification, suppress irrelevant characteristics such as color and background noise, particularly for fine cracks in complex backgrounds. This approach aims to enhance the ability of the model to focus on critical crack features, ultimately improving the recognition accuracy of the model. Meanwhile, this study explores the tracking and identification of crack tips across diverse lithological rock types and under a variety of boundary conditions. The crack tracking model of rock can monitor in real time the initiation, development and expansion of cracks and quantify the impact of fractures on the mechanical properties of rocks. The establishment of the crack tracking model is conducive to analyzing the evolution law of cracks, capturing the subtle changes of cracks. The crack tracking model can be effectively utilized for analyzing the characteristics of rock cracks in complex environments, offering a novel reference for the prevention and mitigation of geological disasters.
